# 5-Hydroxytryptamine G-Protein-Coupled Receptor Family Genes: Key Players in Cancer Prognosis, Immune Regulation, and Therapeutic Response

**DOI:** 10.3390/genes15121541

**Published:** 2024-11-28

**Authors:** Simeng Liu, Mingang He, Hefen Sun, Yi Wu, Wei Jin

**Affiliations:** 1 Key Laboratory of Breast Cancer in Shanghai, Fudan University Shanghai Cancer Center, Shanghai 200032, China; 2 Department of Oncology, Shanghai Medical College, Fudan University, Shanghai 200032, China

**Keywords:** *HTGPCR* family genes, prognosis, pan-cancer, tumor microenvironment, therapeutic targets

## Abstract

Background: Firstly, 5-hydroxytryptamine G-protein-coupled receptors (*HTGPCRs*) are a family of 13 genes associated with cancer progression. Nevertheless, a comprehensive understanding of *HTGPCRs* in cancer remains largely lacking. Method: We tested the gene expression levels and prognostic values for the *HTGPCRs* in relation to pan-cancer. A subsequent analysis examined the relationships among *HTGPCR* expression and clinical characteristics, immune subtypes, stemness scores, tumor microenvironments (TMEs), single-cell analyses, and drug sensitivity. Result: A significant difference in *HTGPCR* expression was found between normal tissues and tumors. *HTR1D/2C* expressed higher levels in breast invasive carcinoma (BRCA), colon adenocarcinoma, and liver hepatocellular carcinoma. *HTGPCR* gene expression was correlated with prognosis in many cancers. *HTR1D/2C* were associated with poorer overall survival for head and neck squamous cell carcinoma. In addition, *HTGPCR* expression correlated significantly with the stemness scores of RNA and DNA, TMB, and MSI, as well as stromal and immune scores of pan-cancer patients. Additionally, the expression of *HTR2A/2B/7* was correlated significantly with immune cells and immune checkpoint genes in a variety of cancers, such as BRCA, brain lower-grade glioma, and lung adenocarcinoma. Immune regulation and TME were both regulated by *HTGPCRs*. Using single-cell analysis, we found that the gene set of *HTGPCRs* correlated with many cancer-related functional states in retinoblastoma. Moreover, drug sensitivity and *HTR4* were significantly correlated. Furthermore, we validated results in breast cancer and found knockdown of *HTR1D* inhibited breast cancer cell growth and metastasis. Conclusion: As prognostic indicators, *HTGPCRs* hold considerable promise and offer insights into the therapeutic targets for malignancy.

## 1. Introduction

As reported by the Global Cancer Statistics 2022, cancer continues to be a major worldwide health issue, with nearly 20 million new cancer diagnoses and approximately 9.7 million cancer-related deaths in 2022 [1,2]. The nervous system and cancer can interact with each other, a long-described phenomenon [3,4]. The roles of the central and peripheral nervous systems are closely linked to cancer, influencing tumor growth, vascular systems, immune responses, and the body’s capacity for resilience and self-repair [5]. Focusing on the interactions between the nervous system and cancer could become a cornerstone of cancer treatment, complementing conventional methods like surgery, radiation, chemotherapy, and immunotherapy [6].

Serotonin (5-HT) is a crucial neurotransmitter in the nervous system, functioning by stimulating a diverse group of serotonin receptors, and is involved in various neurological, psychological, and chronic conditions. Additionally, 5-hydroxytryptamine G-protein-coupled receptors (*HTGPCRs*) belong to the Class A GPCR family and transmit 5-HT signals [7]. The *HTGPCR* gene family is categorized into three groups: Gi protein-coupled receptors (*HTR1A*, *HTR1B*, *HTR1D*, *HTR1E*, *HTR1F*), Gs protein-coupled receptors (*HTR4*, *HTR6*, *HTR7*), and Gq protein-coupled receptors (*HTR2A*, *HTR2B*, *HTR2C*, *HTR5A*, *HTR5BP*) [8]. These receptors are extensively distributed across the body [9]. Recently, there has been a growing focus on the involvement of *HTGPCRs* in cancer development, with studies emerging from various cancer types, such as breast cancer [10], lung cancer [11], gastric cancer [12], colorectal cancer [13], cholangiocarcinoma [14], glioma [15], and prostate cancer [16]. The expression level of *HTR1A/1B/2A/2B/2C/4* and *HTR7* was significantly negatively related to highly malignant breast cancer types [10]. Among HCC cancer patients, the expression of 5-HT1B and 5-HT2B receptors was associated with increased Ki67 and correlated with the size of the tumor [17]. *HTR1D/2A/2B/3A/7* in gastric cancer (GC) might represent a target for the prevention and treatment of GC [18]. Meanwhile, *HTGPCRs* also play an important role in the immune system. *HTR1A* is expressed on various immune cells, the stimulation of the *HTR1A* on T-cells was shown to increase cell survival and cell cycle transition [19], and *HTR2A* is highly expressed in activated T-cells [20]. In the past decade of research, serotonin receptors have been the main targets of a variety of psychiatric drugs [20]. Recently, researchers have gradually begun to explore the application of *HTGPCR* target drugs in cancer [21]. Numerous in vitro studies have demonstrated the antitumor effects of 5-HT receptor antagonists in varieties of cancer, including colorectal cancer [22], gastric cancer [23], pancreatic cancer [24], and breast cancer [25]. Despite the fact that people have a comprehensive understanding of the structure of *HTGPCRs* and their roles in the nervous system, systematic studies of *HTGPCRs* in human tumors have not been reported.

This research utilized TCGA cancer datasets to explore the particular relationship between *HTGPCRs* and 33 different types of cancer. This study aims to determine the potential role and distinct prognostic significance of *HTGPCRs* as biomarkers in cancer diagnosis and therapy. Assessing the varied expression, survival rates, and immune infiltration of different *HTGPCR* subtypes across multiple cancers offers insights for novel anti-cancer approaches.

## 2. Materials and Methods

### 2.1. Data Gathering

The UCSC Xena website (http://xena.ucsc.edu/ (accessed on 3 June 2024)) provided us with all TCGA pan-cancer data, including gene expression, survival, phenotypic, stemness, and immune subtypes. The types of cancers included in the TCGA pan-cancer data are shown in Table S1.

Information on BRCA, encompassing mutations, RNA sequencing expression data (Level 3), and associated clinical information, was sourced from the TCGA database (https://portal.gdc.cancer.gov (accessed on 3 June 2024)). The Genotype-Tissue Expression (GTEx) datasets were available online at http://www.gtexportal.org/home/datasets/ (accessed on 3 June 2024).

### 2.2. Expression Analysis

Utilizing Perl scripts, we retrieved the expression levels of *HTGPCRs* and examined the differences in these levels across 33 various cancer types. Furthermore, the expression patterns of *HTGPCR* family genes were depicted through a box plot and heatmap generated with the R packages ‘ggpubr’ and ‘heatmap’.

By using the STRING database, we constructed a protein–protein interaction (PPI) network. The relationships among *HTGPCR* family genes were analyzed using the ‘corrplot’ package in R.

### 2.3. Survival Analysis

By employing the Kaplan–Meier method and log-rank test, we investigated the link between *HTGPCR* expression and cancer prognosis across various TCGA datasets using the ‘survival’ R package. A Cox analysis was also performed to evaluate the relation between *HTGPCR* expression and overall survival across various cancers. Forest plots were generated using R packages ‘forestplot’ and ‘survival’.

Moreover, we evaluated the relationship between *HTGPCR* expression and overall tumor prognosis using the Kaplan–Meier Plotter online tool (https://kmplot.com/analysis/ (accessed on 20 may 2024)). Kaplan–Meier plots from the TCGA, GEO, and EGA databases were utilized to evaluate *HTGPCRs* in patients suffering from 21 different types of tumors. Differential analysis was also utilized to determine differences between disease stages in *HTGPCR* expression levels.

### 2.4. Gene Alteration in Pan-Cancer

The cBioPortal for cancer genomics (http://www.cbioportal.org (accessed on 20 May 2024)) offers an extensive collection of information, including somatic mutations, copy number alterations, gene expression profiles, DNA methylation patterns, and protein enrichment data. This research investigated if changes in *HTGPCRs* influenced patient survival rates, using data from the ICGC/TCGA Pan-Cancer Analysis of Whole Genomes Consortium 2020. Furthermore, the analysis examined variations in the copy number variation (CNV) of various cancer types as well as variations in single nucleotide variation (SNV) [26].

### 2.5. Correlation Between HTGPCR Expression, Immune Subtype, and Tumor Microenvironment

Through Spearman correlation, 33 tumor samples were analyzed for their RNA stemness score (RNAss), DNA stemness score (DNAss), microsatellite instability (MSI), tumor mutational burden (TMB), tumor purity, and expression of *HTGPCRs*. Moreover, scores for stromal and immune cells across various tumor types were determined utilizing the R packages ‘estimate’ and ‘limma’. Based on the TCGA database, the association between *HTGPCR* expression and immunological subtypes of cancer was also examined.

### 2.6. Correlation Analysis of HTGPCRs and Immunotherapy Outcome

In 33 TCGA tumors, ssGSEA was used to investigate the potential association between *HTGPCRs* and immune cell infiltration. The Spearman correlation test was used to determine whether there was a correlation between immune checkpoint expression (PD-L1, PD-1, and CTLA4) and *HTGPCR* expression in 33 TCGA tumors. A heatmap was generated using the R package “pheatmap”.

### 2.7. Single-Cell Analysis

Based on the IMMUcan SingleCell RNAseq Database (https://immucanscdb.vital-it.ch/ (accessed on 3 June 2024)), we examined the expression of *HTGPCRs* in various cancer single-cell datasets. Additionally, we explored the CancerSEA database (http://biocc.hrbmu.edu.cn/CancerSEA/home.jsp (accessed on 3 June 2024)) to determine whether there is a connection between the *HTGPCR* gene set and 14 functional states related to cancer. CancerSEA is a versatile site designed to thoroughly investigate various functional states of individual cancer cells, encompassing 14 cellular activities, including cell cycle, angiogenesis, apoptosis, metastasis, DNA repair, differentiation, DNA damage, quiescence, EMT, hypoxia, inflammation, invasion, proliferation, and stemness [27].

### 2.8. Nomogram and Calibration Curve Construction

The nomogram has been extensively used for prognosticating cancer outcomes [28]. Its accuracy was measured using a calibration curve [29]. *HTGPCRs* were included in the construction of the nomogram prediction model and the calibration curve was drawn to evaluate the model calibration with the R “rms” package.

### 2.9. Drug Sensitivity Analysis

A database called CellMiner (https://discover.nci.nih.gov/cellminer/ (accessed on 3 June 2024)) collects data on the molecular expression of 60 different human cancer cell lines [30]. Our study evaluated the correlation between *HTGPCR* family members’ expression and drug sensitivity by using the CellMiner database, as well as using TISIDB’s drug module to analyze the drugs targeting these genes.

### 2.10. Cell Line

The HEK-293T, MDA-MB-231, and LM2 were obtained from the American Type Culture Collection (ATCC). Cell lines were cultured in DMEM culture media with 1% Penicillin/Streptomycin and 10% fetal bovine serum (FBS; Gibco), maintained at 37 °C and 5% CO_2_.

MDA-MB-231 and LM2 *HTR1D*ko cells were generated by CRISPR/cas9-mediated genome engineering. The sequence information was *HTR1D* sgRNA_1 (5′-CAT-CAC-CGA-ATA-AGACT-3′), *HTR1D* sgRNA_2 (5′-TAC-AGT-AAA-CGC-AGG-ACG-GC-3′), sg_nc (5′-CAC-CGT-TCG-GCT-GGT-GTG-CGT-TCAC-3′). These CRISPR guide RNAs were cloned into the LentiCRISPR v2 plasmid (Addgene, 52961) and cell transfection and selection procedures were described previously [31].

### 2.11. Cell Proliferation and Colony Formation Assays

For cell proliferation assays, cells were plated into 96-well plates for 2000 cells per well and cell viability assessments were conducted by the CCK8 assay (Vazyme) for 7 days. For colony formation assays, 200 cells were plated into 12-well plates in triplicate and cultured for 10 days. Colonies were fixed and stained with 0.5% crystal violet and colony numbers were counted.

### 2.12. Wound-Healing, Cell Migration, and Invasion Assays

For wound-healing assays, cells were seeded in six-well plates and scratches were created after 24 h of incubation. Following this, cells were washed with PBS and incubated in a medium containing 0.1% FBS for the specified times. For migration and invasion assays, 100,000–200,000 cells in serum-free medium were added to the upper chambers, which were coated with growth-factor-reduced Matrigel (for invasion) or left uncoated (for migration) (BD Biosciences, Franklin Lakes, NJ, USA). After 5 or 12 h, cells on the lower membranes of the transwell chambers were fixed in methanol, stained with 0.5% crystal violet, and counted under a microscope.

### 2.13. Statistical Analysis

The statistical analyses were conducted using R version 4.2.1. To evaluate the differences between the two groups, either the student’s t-test or analysis of variance (ANOVA) was applied. Correlation analyses were performed using the Spearman correlation coefficient. The false discovery rate was calculated using the Benjamini–Hochberg procedure. The prognostic significance of *HTGPCR* expression in each malignancy was assessed through the Kaplan–Meier method and univariate Cox regression analysis. All experiments were conducted in triplicate. Additionally, *p* < 0.05 was considered statistically significant and indicated as follows: * *p* < 0.05, ** *p* < 0.01, *** *p* < 0.001, and **** *p* < 0.0001.

## 3. Results

### 3.1. Expression of HTGPCRs in Pan-Cancer

**Figure 1:**
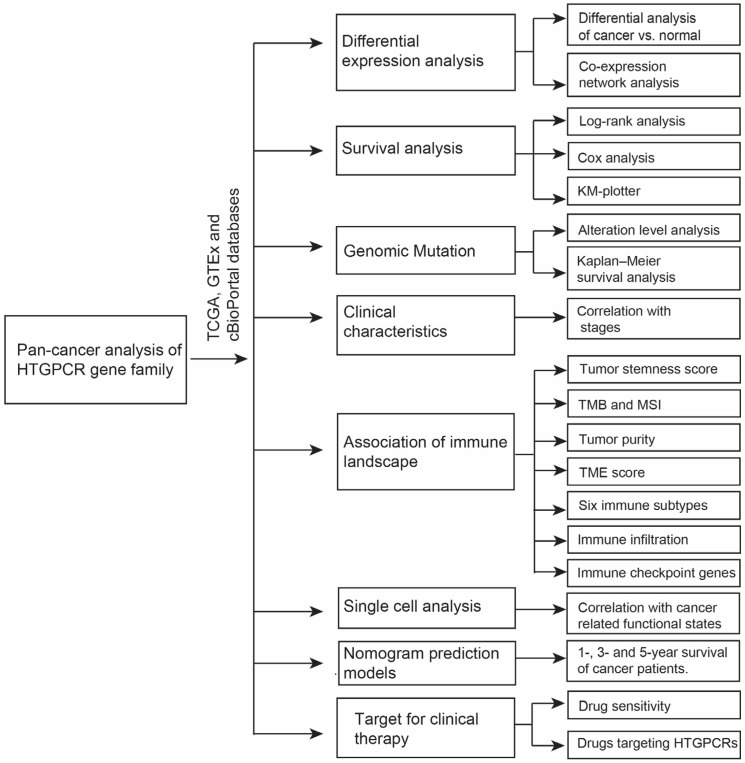
The flow chart of the pan-cancer analysis of the *HTGPCR* gene family.

**Figure 2:**
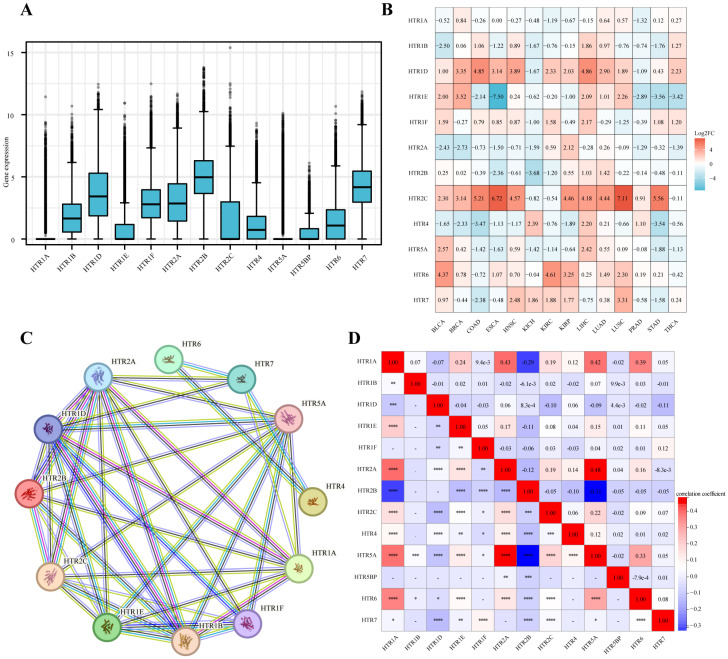
Gene expression levels of the *HTGPCR* family and their correlation in various cancer types from TCGA. (**A**) Boxplot showing *HTGPCR* expression in pan-cancer. (**B**) Heatmap displaying variations in *HTGPCR* expression across various cancer types. The orange and blue indicate the high or low expression, respectively. (**C**) The PPI network of *HTGPCRs*. (**D**) The correlation between *HTGPCRs*. The red dot indicates a positive correlation and the blue dot indicates a negative correlation. * *p* < 0.05, ** *p* < 0.01, *** *p* < 0.001, ****p < 0.0001.

A detailed flow chart of this study is provided in Figure 1. The TCGA database analysis revealed the expression of *HTGPCRs* across various cancers. Specifically, *HTR1D/2B/7* exhibited high levels of expression while *HTR1B/1F/2A/2C/4/6* demonstrated moderate expression. Conversely, *HTR1A/1E/4/5A/5B* were found to be expressed less in a pan-cancer context (Figure 2A). Detailed examination showed that *HTR1D* was particularly overexpressed in liver hepatocellular carcinoma (LIHC) and *HTR2C* showed a similar pattern in lung squamous cell carcinoma (LUSC) (Figure 2B). Further insights were provided by the protein–protein interaction (PPI) network of *HTGPCRs*, sourced from the STRING database, which illustrated the intricate interactions among *HTGPCRs* (Figure 2C). Notably, *HTR2A* and *HTR5A* displayed a robust positive correlation, suggesting a potential synergistic relationship. In contrast, a strong negative correlation was observed between *HTR2B* and *HTR5A*, indicating a possible antagonistic interaction (Figure 2D).

**Figure 3:**
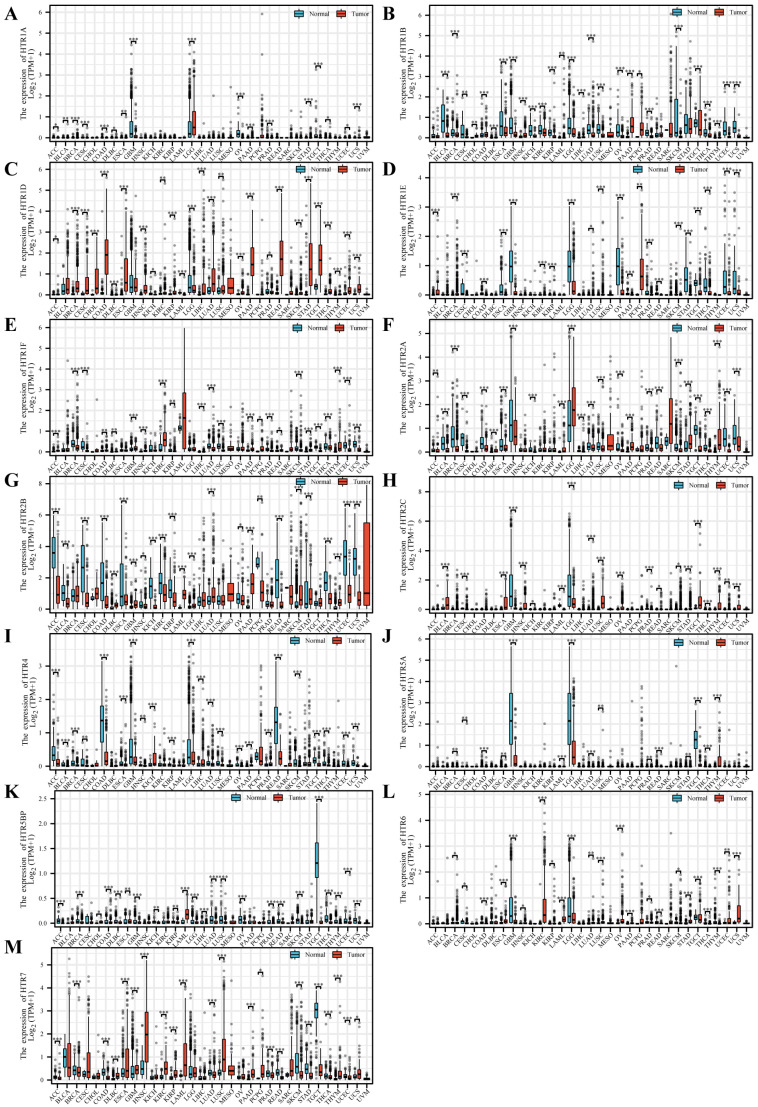
Expression levels of the *HTGPCRs* in different types of cancer and in normal tissues from TCGA and GTEx databases. (**A**) *HTR1A* (**B**) *HTR1B* (**C**) *HTR1D* (**D**) *HTR1E* (**E**) *HTR1F* (**F**) *HTR2A* (**G**) *HTR2B* (**H**) *HTR2C* (**I**) *HTR4* (**J**) *HTR5A* (**K**) *HTR5BP* (**L**) *HTR6* (**M**) *HTR7*. The blue boxplots indicate the normal tissues and the red boxplots indicate the cancer tissues. * *p* < 0.05, ** *p* < 0.01, *** *p* < 0.001.

Next, we used TCGA-GTEx data to assess the different expressions of *HTGPCRs* between normal tissues and 33 malignancies (Table S1). The result indicated that *HTR1A* was expressed at low levels in ovarian serous cystadenocarcinoma (OV) and glioblastoma multiforme (GBM) while being highly expressed in brain lower grade glioma (LGG) (Figure 3A). *HTR1B* was lowly expressed in esophageal carcinoma (ESCA), GBM, kidney renal clear cell carcinoma (KIRC), kidney chromophobe (KICH), kidney renal papillary cell carcinoma (KIRP), brain lower grade glioma (LGG), lung squamous cell carcinoma (LUSC), lung adenocarcinoma (LUAD), OV, prostate adenocarcinoma (PRAD), thyroid carcinoma (THCA), skin cutaneous melanoma (SKCM), uterine carcinosarcoma (UCS), and uterine corpus endometrial carcinoma (UCEC) and highly expressed in colon adenocarcinoma (COAD), pancreatic adenocarcinoma (PAAD), head and neck squamous cell carcinoma (HNSC), pheochromocytoma and paraganglioma (PCPG), and testicular germ cell tumors (TGCT) (Figure 3B). HIR1D was highly expressed in different kinds of cancer, including BRCA, CHOL, CESC, ESCA, COAD, KIRP, LUAD, LIHC, LUSC, pancreatic adenocarcinoma (PAAD), rectum adenocarcinoma (READ), stomach adenocarcinoma (STAD), testicular germ cell tumors (TGCT), THCA, and UCEC, while being lowly expressed in LGG and PRAD (Figure 3C). *HTR1E* was found to be lowly expressed in CESC, GBM, ESCA, OV, LGG, PRAD, READ, STAD, SKCM, TGCT, THCA, UCS, and UCEC and highly expressed in adrenocortical carcinoma (ACC) (Figure 3D). *HTR1F* was highly expressed in KIRC, acute myeloid leukemia (LAML), and thymoma (THYM) and lowly expressed in BRCA CESC, LUAD, LUSC, UCEC, and UCS (Figure 3E). *HTR2A* was lowly expressed in bladder urothelial carcinoma (BLCA), BRCA, CESC, COAD, ESCA, GBM, LUAD, LUSC, OV, PRAD, READ, SKCM, TGCT, THCA, UCEC, and UCS while being highly expressed in LGG, PAAD, STAD, SARC, and THYM (Figure 3F). *HTR2B* was lowly expressed in different kinds of cancers, including ACC, BLCA, COAD, CESC, ESCA, KIRC, KICH, KIRP, READ, PCPG, STAD, THCA, UCEC, and UCS, and highly expressed in GBM, LUAD, LAML, and PAAD (Figure 3G). *HTR2C* was lowly expressed in GBM and LGG and highly expressed in BLCA, ESCA, LUSC, TGCT, and THYM (Figure 3H). *HTR4* can be found to be lowly expressed in ACC, CESC, COAD, GBM, LGG, and READ and highly expressed in KICH and LIHC (Figure 3I). HTR5 A was lowly expressed in GBM, LGG, and TGCT while being highly expressed in THYM (Figure 3J). *HTR5BP* was lowly expressed in TGCT and highly expressed in LAML (Figure 3K). *HTR6* was lowly expressed in GBM and LGG while being highly expressed in KIRC, LAML, and UCS (Figure 3L). *HTR7* was found to be highly expressed in different kinds of cancer, including BLCA, BRCA, CESC, HNSC, ESCA, KIRC, LAML, KIRP, LUSC, PAAD, and PCPG, and lowly expressed in PRAD, READ, SKCM, and TGCT (Figure 3M). The expression level of *HTGPCRs* in different tumors showed the specificity of genes and tumors, for example, *HTR1B* and *HTR2B* were lowly expressed in most cancers while *HTR1D* and *HTR7* were highly expressed in most cancers. These findings showed the complex regulatory networks within the *HTGPCRs* and their potential implications in cancer biology and therapeutic strategies.

### 3.2. Clinical Prognostic Analysis of HTGPCRs in Pan-Cancer

We performed an extensive assessment using several databases to determine the prognostic significance of *HTGPCRs* in different types of cancer (Table 1). Kaplan–Meier survival plots demonstrated a significant link between *HTGPCR* expression levels and patient outcomes in specific types of cancer. The high expression of *HTR1A* was associated with better overall survival (OS) in LGG and GBMLGG (Figure S1A). High expression of *HTR1B* was linked to poor OS in KIRP and MESO while high expression of *HTR1B* was related to better OS in ESCC, KIRC, and READ (Figure S1B). Furthermore, the findings indicated a link between poor overall survival and elevated *HTR1D* levels in individuals with BLCA, GBMLGG, HNSC, LGG, LIHC, LUAD, LUADLUSC, PAAD, and SARC (Figure S1C). *HTR1E* proved useful throughout GBMLGG (Figure S1D). High expression of *HTR1F* was associated with poor OS in LAML, LGG, GBMLGG, MESO, and STAD (Figure S1E). High expression of *HTR2A* in pan-cancer was mainly related to poor OS in KICH and LUSC while being related to better OS in GBMLGG and LGG (Figure S2A). *HTR2B* was found to be a protective factor in ESAD, ESCA, LUAD, PCPG, and UCEC while being a risk factor in COAD, GBMLGG, LUADLUSC, LUSC, STAD, and UVM (Figure S2B). The OS rates were negatively influenced by *HTR2C* in HNSC and STAD, whereas a positive impact was observed in GBM and GBMLGG (Figure S2C). *HTR4* was related to better OS in BLCA, LUAD, and SKCM (Figure S2D). In GBMLGG, the expression of *HTR5A* was positively related to OS (Figure S2E). *HTR5BP* was found to be a protective factor in LUADLUSC while being a risk factor in LGG (Figure S2F). The expression of *HTR6* was negatively related to the OS in ACC, GBM, KIRP, MESO, and UCEC while being positively related to OS in GBMLGG and LGG (Figure S2G). Furthermore, we found that *HTR7* can be a risk factor in GBMLGG, GBM, LAML, and STAD while being a protective factor in KICH (Figure S2H). Specifically, the high expression of *HTGPCR* genes related to a high survival rate while, in some cancers, high expression of *HTGPCR* was associated with poor prognosis, which indicated a complex and variable impact of these genes’ expression on cancer prognosis. Owing to the high expression of the *HTGPCR* gene family in the brain, the *HTGPCR* gene family was closely related to the prognosis of patients with glioma.

In our comprehensive analysis utilizing Cox regression, most of the genes in the *HTGPCR* family were found to be high-risk factors for KICH, except *HTR4*. Additionally, *HTR1A/1B/1D/1E/1F/2A/2B/4* were high-risk factors for DLBC, *HTR1A/1E/1F/2B/4* were high-risk factors for TGCT, and *HTR1A/1B/1D/6* were high-risk factors for THYM (Figure S3, Table 1).

In order to further elucidate the prognostic significance of *HTGPCR* family genes in a pan-cancer setting, a validation study was performed utilizing the Kaplan–Meier plotter. The results of this analysis confirmed and strengthened our findings. The expressions of *HTR1B* and *HTR1F* are negatively related to KIRP (Figure S4A,D). *HTR1D/2C/5A/7* were negatively related to the OS in LIHC and KIRC (Figure S4B,G,I,K). Meanwhile, *HTR1E* and *HTR4* were positively related to the OS in BLCA (Figure S4C,H). The expression of *HTR1E* and *HTR6* related to the poor OS in UCEC (Figure S4C,J).

### 3.3. Genomic Changes of HTGPCR Across Pan-Cancer

**Figure 4:**
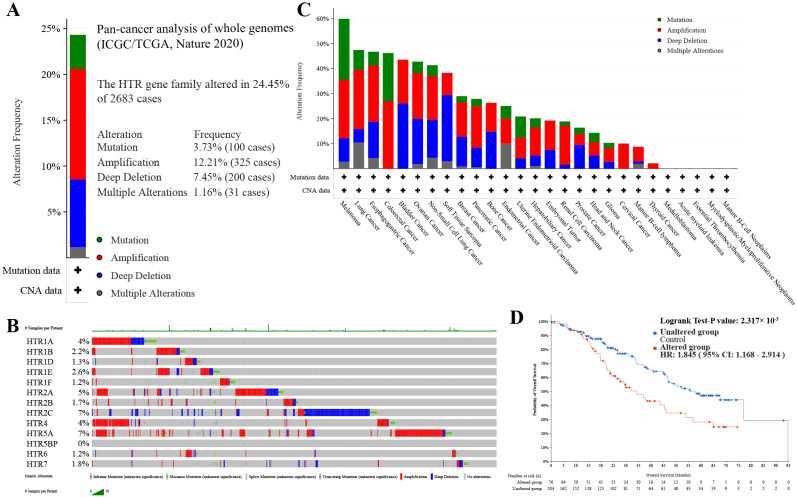
The *HTGPCR* family alteration in various cancers was identified using the cBioPortal database. (**A**) Alteration frequency of the *HTGPCR* family at the overall level in pan-cancer. (**B**) *HTGPCR* family members’ alteration level in different types of cancers. (**C**) A visual display of genomic alteration based on the *HTGPCR* genes. (**D**) Kaplan–Meier curve comparing overall survival in cases with and without the alterations of *HTGPCR* genes.

During our extensive exploration of the *HTGPCR* gene family in different types of cancers, we have identified notable genomic changes within its members. By utilizing the cBioPortal database, we conducted a thorough evaluation of mutations, copy number variations (CNVs), and single nucleotide variants (SNVs) within the *HTGPCR* family from a pan-cancer perspective. Our integrative analysis, which encompassed 2683 whole-cancer genomes and their corresponding normal tissues across 38 tumor types, revealed that the *HTGPCR* gene family was altered in 24.45% of the 2658 cases studied. The alterations were distributed among various *HTGPCR* genes, with *HTR1A* showing a 4% alteration rate, *HTR1B* at 2.2%, *HTR1D* at 1.3%, and so forth, culminating in *HTR7* with a 1.8% alteration rate (Figure 4A,B).

The predominant types of genomic alterations observed were amplifications, occurring in 12.21% of the cases (325 instances), and deep deletions, found in 7.45% of the cases (200 instances). These genomic events were detectable across a spectrum of cancers, including melanoma, lung cancer, colorectal cancer, esophagogastric cancer, ovarian cancer, bladder cancer, and non-small-cell lung cancer, among others. Deep deletions, an equally significant class of mutation for the *HTGPCR* family in pan-cancer, were also identified in cancers such as bladder cancer, ovarian cancer, non-small-cell lung cancer, soft tissue sarcoma, and more (Figure 4C). Furthermore, the presence of *HTGPCR* alterations in pan-cancer patients correlated with a poorer prognosis (Figure 4D). As depicted in Figure S5, diploid and shallow deletion were the primary alterations in pan-cancer, which were associated with the overexpression of *HTR1B/1D/1F/2A/7*.

### 3.4. Correlation Among Clinical Characteristics and HTGPCR Family Gene Expression in Pan-Cancer

We then utilized the TCGA database to investigate the association between the expression of *HTGPCR* family members and various cancer stages. The mRNA expressions of *HTR1D* in LUAD, KIRP, and KIRC were significantly higher in Stages I and III than in the other stages while, in LIHC and THCA, the expression of Stage I was relatively lower than in the others (Figure S6C). The expression of *HTR1F* in KIRP was positively correlated with staging. In ESCA, STES, and STAD (Figure S6E), the expression of *HTR2A* was relatively higher in Stage II and Stage III patients (Figure S6F). The expression of *HTR2B* in PAAD was positively correlated with its stage while being negatively correlated with the stage in TGCT and BLCA (Figure S6G). Based on the above results, it can be concluded that the expression of *HTGPCR* family genes correlated with TNM staging.

### 3.5. Correlation Among HTGPCR Family Gene Expression and Stemness Score and TME in Pan-Cancer

**Figure 5:**
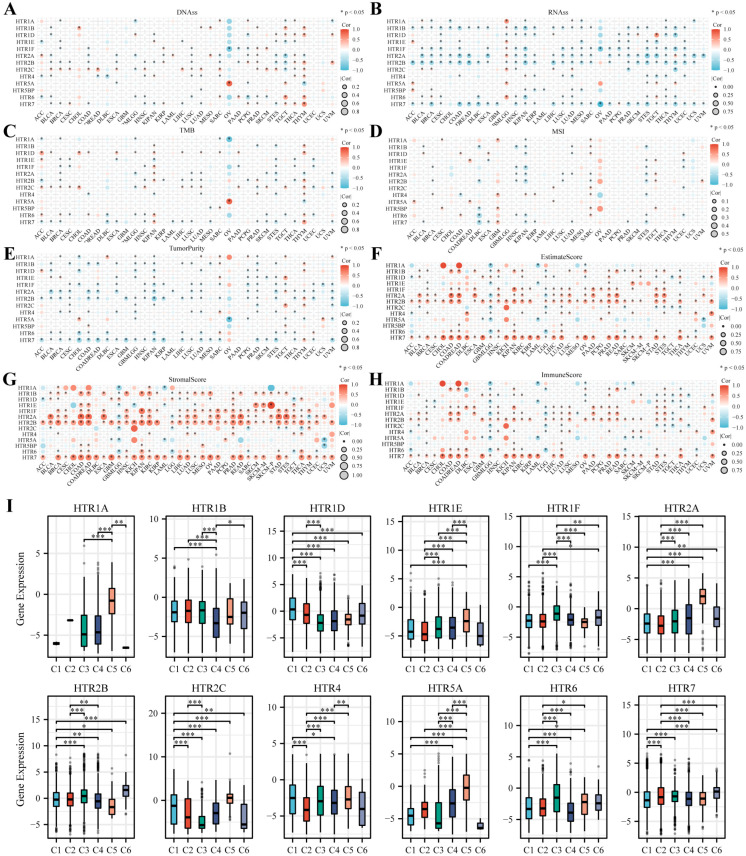
The relationship between *HTGPCR* family genes and various factors, including (**A**) DNAss, stemness scores based on DNA-methylation; (**B**) RNAss, stemness scores based on mRNA; (**C**) TMB, tumor mutation burden; (**D**) MSI, microsatellite instability; (**E**) tumor purity; (**F**) estimate score; (**G**) stromal score; (**H**) immune score; and (**I**) the relationship between the expression of *HTGPCR* family genes and six distinct immune subtypes in pan-cancer within the TCGA dataset. C1, wound healing; C2, IFN-γ dominant; C3, inflammatory; C4, lymphocyte depleted; C5, immunologically quiet; C6, TGF-β dominant. * *p* < 0.05, ** *p* < 0.01, *** *p* < 0.001.

Stemness and TME play crucial roles in cancer research and were found to be connected with the mechanisms, development processes, and treatment responses of cancer. In this context, we investigated the relationship between *HTGPCR* family gene expression and various aspects of stemness and TME. By investigating the correlation between the *HTGPCRs* and stemness, different *HTGPCR* family members were linked to RNAss, DNAss, TMB, and MSI in various cancer types. A negative correlation was found between the *HTGPCR* family and the RNAss in pan-cancer, except for GMBLGG (Figure 5B). Other indicators, such as the DNAss, TMB, and MSI, varied in different types of cancer (Figure 5A,C,D). Analysis of TME-related scores showed that most *HTGPCR* expressions were strongly negatively correlated with tumor purity and significantly positively correlated with the estimate, stromal, and immune scores (Figure 5E–H).

Scientists investigated the immune landscape across various types of cancer and ultimately classified them into six immune subtypes (C1–C6), including C1 (wound healing), C2 (IFN-γ dominant), C3 (inflammatory), C4 (lymphocyte deficient), C5 (immunologically silent), and C6 (TGF-β dominant), which were assumed to define the immune response with implications for further exploration of immunotherapy [32]. Our finding indicated that the expression of all *HTGPCR* gene family members showed a significant connection with C1–C6 (Figure 5 I). Additionally, C5 and the high expression of *HTR5A* were tightly related, indicating that *HTR5A* may have a cancer-suppressing function, and *HTR7*’s high expression was related to C2, which indicated that *HTR7* related to a high level of immune infiltration and intertumoral heterogeneity. These insights underscore the multifaceted roles of *HTGPCR* genes in the modulation of cancer stemness, TME, and immune responses, offering a rich avenue for further research into targeted therapies and immunotherapies.

### 3.6. Immune Status of HTGPCR Family Genes in Pan-Cancer

**Figure 6:**
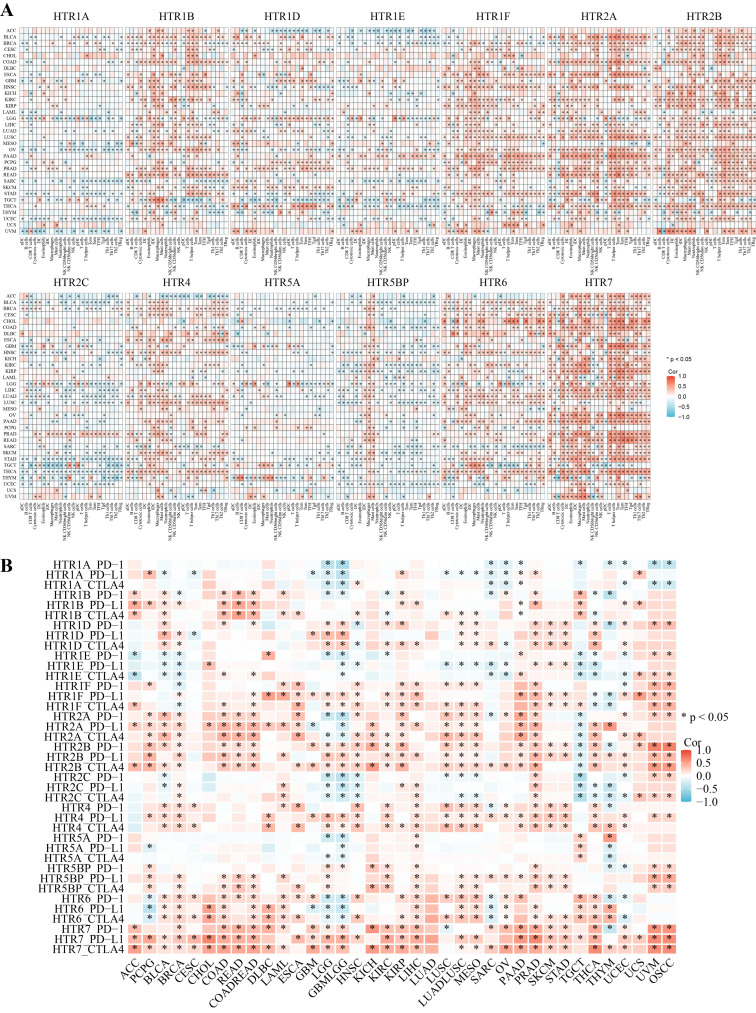
(**A**) Correlation between gene expression of each *HTGPCR* and immune cell infiltration level estimated by ssGSEA algorithms across 33 cancer types. (**B**) Correlation between *HTGPCR* gene expression levels and the mRNA expression of three common immune checkpoints in various tumor types. Red, positive correlation; Blue, negative correlation. * *p* < 0.05.

To further explore the relationship between the *HTGPCR* family and tumor immunity, we evaluated the relationship between immune cell infiltration and *HTGPCR* expression using ssGSEA. *HTGPCRs* were significantly associated with a large number of immune cells. In most tumors, the expression of *HTR1A/1E/2C/5A* was negatively correlated with immune cell infiltration. Meanwhile, *HTR2A/2B/7* were strongly positively correlated with most immune cell infiltration, such as T cells, DCs, macrophages, neutrophils, and so on. Moreover, the correlation between *HTR1B/1D/1E/1F/4/5A/5BP* and immune cell infiltration varied across different types of cancer (Figure 6A).

Nowadays, immune checkpoint inhibitors have been widely used in the treatment of a variety of cancers and the application of immune checkpoints provides new treatment options for cancer and other diseases [33,34]. Therefore, we analyzed the correlation between the *HTGPCR* gene family and the main immune checkpoint genes (PD-1, PD-L1, and CTLA4). In pan-cancer, high expression of *HTR1B/1F/2B/4/7* was positively correlated with the expression of PD-1, PD-L1, and CTLA4 while the expression of *HTR1A* was negatively correlated with the expression of PD-1, PD-L1, and CTLA4 in GLB, LGG, SARC, OV, and PAAD (Figure 6B). These findings suggest that *HTGPCRs* potentially play a role in modulating T-cell-mediated immune responses, which may be of great importance in the development of novel approaches for tumor immunotherapy.

### 3.7. The Expression of HTGPCRs in Single-Cell Level

**Figure 7:**
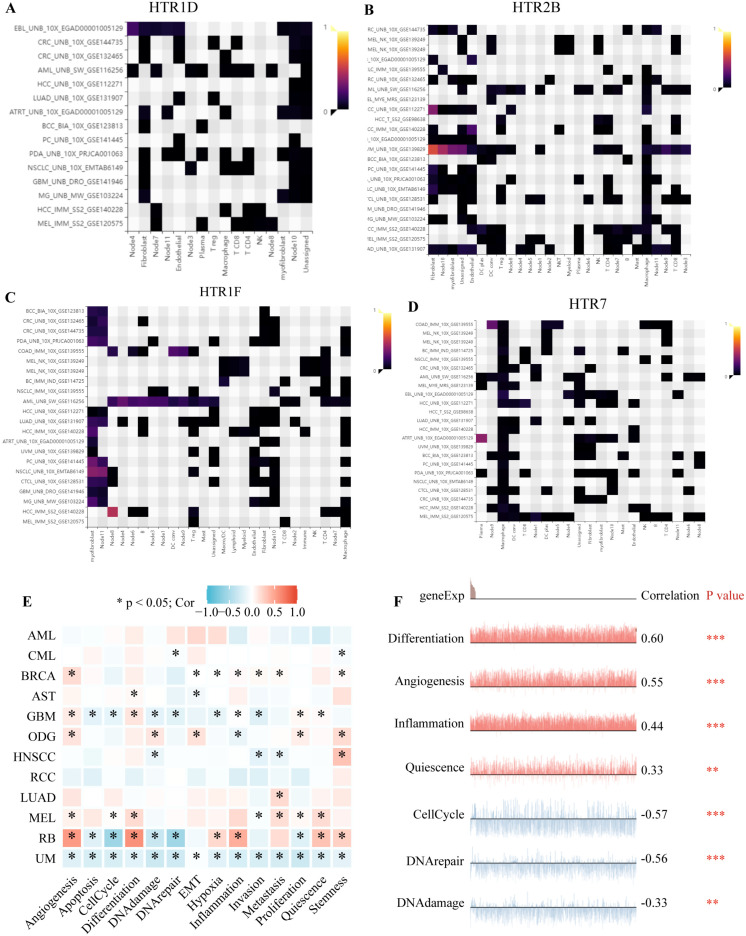
Single-cell analysis. The expression distribution of *HTR1D* (**A**), *HTR2B* (**B**), *HTR1F* (**C**), and *HTR7* (**D**) at the single-cell level. (**E**) The correlation between the gene set of *HTGPCRs* and cancer-related functional states. Red, positive correlation; Blue, negative correlation. (**F**) The correlation between the gene set of *HTGPCRs* and cancer-related functional states in RB. * *p* < 0.05, ** *p* < 0.01, *** *p* < 0.001.

By analyzing various single-cell datasets from different cancer types, we obtained insights into the expression patterns of *HTR1D* (Figure 7A), *HTR1E* (Figure S7A), *HTR1F* (Figure 7C), *HTR2A* (Figure S7B), *HTR2B* (Figure 7B), *HTR4* (Figure S7C), *HTR5BP* (Figure S7D), *HTR6* (Figure S7E), and *HTR7* (Figure 7D) at the single-cell level. *HTR2B* was predominantly highly expressed in fibroblasts and *HTR1F* was relatively highly expressed in myofibroblasts. Through the integration of single-cell datasets, we examined the relationship between the gene set of *HTGPCRs* and cancer-related functional states (Figure 7E), revealing correlations between *HTGPCR* gene sets and various cancers and functional states. Specifically, Figure 7E illustrates the association between *HTGPCR* gene sets and cancer-related functional states in retinoblastoma (RB). Additionally, Figure S7F depicts the expression patterns of the *HTGPCR* gene set using a t-SNE plot in RB.

### 3.8. Construction of Nomogram Prediction Models

**Figure 8:**
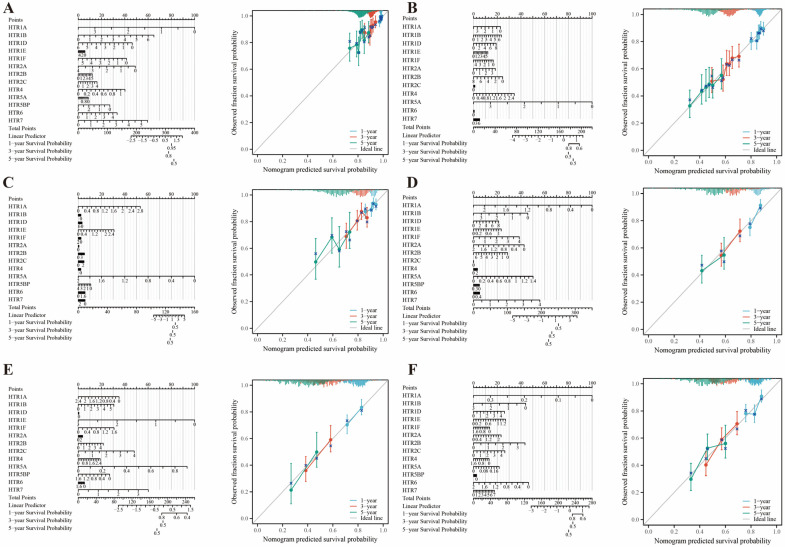
Establishment and calibration curves of nomogram prediction models with 13 *HTGPCR* family genes to predict the 1-, 3- and 5-year survival of cancer patients. (**A**) BRCA. (**B**) LUADLUSC. (**C**) COADREAD. (**D**) LIHC. (**E**) STAD. (**F**) HNSC.

We identified the six most prevalent cancers in the population, including BRCA, LUAD, LUSC, COAD, PRAD, STAD, and LIHC, and then developed novel nomogram models for these cancers. Utilizing 13 genes from the *HTGPCR* family, these models demonstrated robust predictive accuracy for patient survival at 1, 3, and 5 years (Figure 8A–F). Calibration plots showed a strong agreement between the calibration curves and the ideal reference line, underscoring the high accuracy and predictive strength of our models. These models offer a valuable resource for creating personalized treatment strategies, with the potential to significantly improve patient outcomes.

### 3.9. Drug Sensitivity Analysis

**Figure 9:**
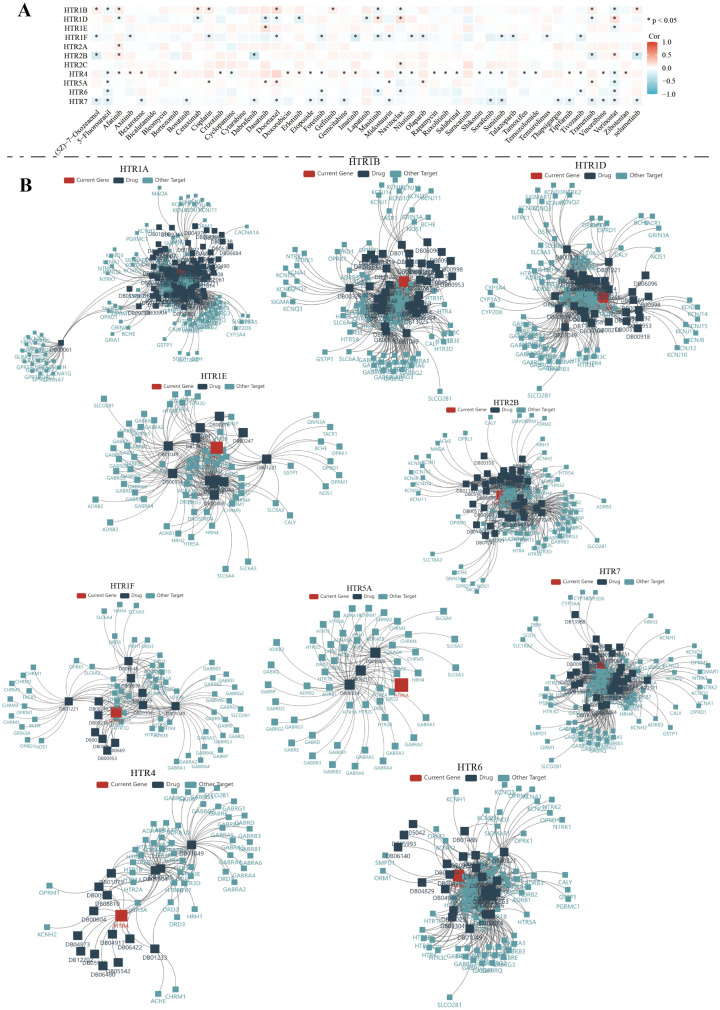
(**A**) Correlation of *HTGPCRs* with sensitivity to common chemotherapeutic agents based on the GDSC database. (**B**) Drugs targeting *HTGPCRs* collected from the DrugBank database. Red, positive correlation; Blue, negative correlation. * *p* < 0.05.

We explored the relationship between the *HTGPCR* family and drug sensitivity using the GDSC database. Our analysis indicated a significant correlation between the expression of *HTGPCR* family genes and drug sensitivity. Specifically, the expression of *HTR1B* was positively correlated with drug sensitivity, including 5z-7-oxozeaenol, afatinib, cetuximab, cisplatin, docetaxel, gefitinib, trametinib, and selumetinib. *HTR1D* showed a negative correlation with the sensitivity of afatinib, cetuximab, dasatinib, docetaxel, erlotinib, lapatinib, and trametinib. The expression of *HTR4* and *HTR7* was found to be correlated with multiple drug sensitivities, including 5z-7-oxozeaenol, 5-fluorouracil, bexarotene, bosutinib, dabrafenib, docetaxel, and others (Figure 9A). Furthermore, the TISIDB database was used to analyze the current drugs targeting *HTGPCRs* in the DrugBank database. Currently, various drugs targeting the *HTGPCR* family genes, mainly focusing on psychotropic drugs, are used for the treatment of depression, anxiety, schizophrenia, and other conditions, including the selective 5-HT receptor agonists zolmitriptan, 5-HT receptor agonist eletriptan, 5-HT1A receptor agonist buspirone, dopamine receptor agonists cabergoline, and ropinirone, etc. (Figure 9B).

### 3.10. Knockdown of HTR1D Inhibits Breast Cancer Cell Growth and Metastasis In Vitro

**Figure 10:**
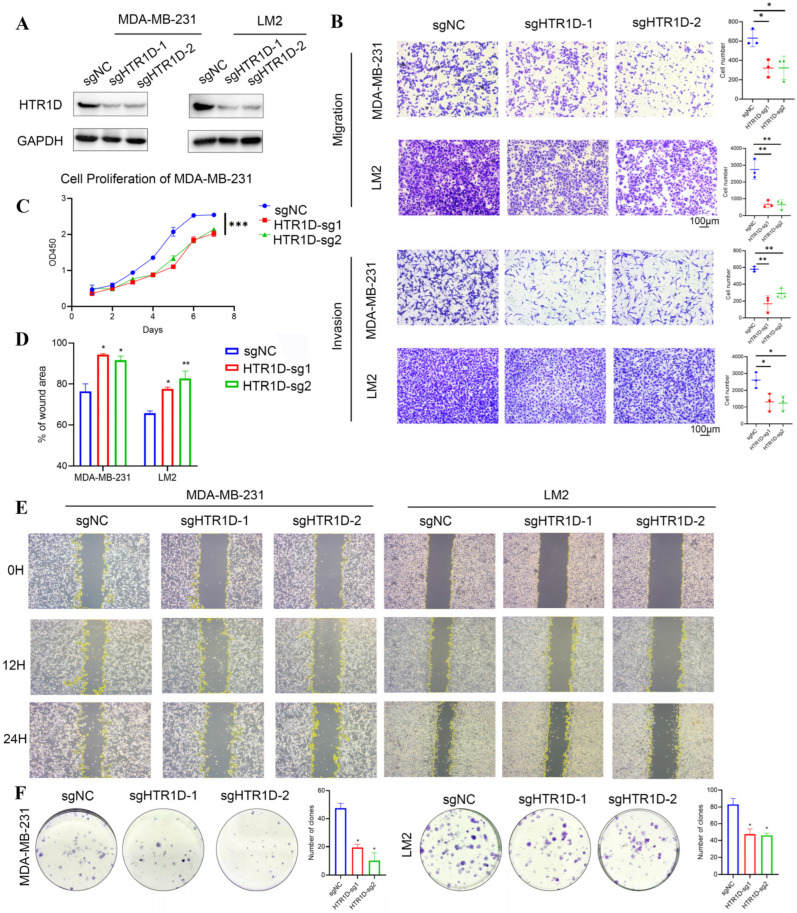
Knockdown of *HTR1D* inhibits breast cancer cell proliferation and metastasis in vitro. (**A**) Immunoblot analysis of *HTR1D* protein level in MDA-MB-231 or LM2 cells transduced with sgRNA targeting *HTR1D* and control. (**B**) The migration and invasion ability in *HTR1D* knockdown cells. (**C**) The proliferation of MDA-MB-231 with *HTR1D* knockdown. (**D**,**E**) Wound healing of cells with *HTR1D* knockdown. (**F**) Colony formation of cells with *HTR1D* knockdown. (Values are expressed as the means ± SDs; * *p* < 0.05, ** *p* < 0.01 and *** *p* < 0.001).

Through the above analysis and previous studies, we found that the HTRGPCR family is closely related to cancer progression. For example, *HTR1D* was highly expressed in a variety of tumors, including BRCA, CHOL, CESC, ESCA, COAD, KIRP, etc. High expression of *HTR1D* was associated with poor prognosis of a variety of tumors. Additionally, there is rarely evidence for the function of *HTR1D* in cancer. Our previous studies focused on breast cancer metastasis research; therefore, we further validated our analysis results in breast cancer cells. We constructed *HTR1D* knockdown cells on MDA-MB-231 and LM2 cells using the CRISPR/cas9 knockdown system (Figure 10A). In cell migration and invasion assays, we found that *HTR1D* knockdown significantly suppressed the migration and invasion ability of MDA-MB-231 and LM2 (Figure 10B) and wound healing assay also confirmed the results (Figure 10 D-E). We also found that HTR1D knockdown significantly inhibited the proliferation of MDA-MB-231 (Figure 10C). In addition, a cloning assay showed that knockdown of *HTR1D* significantly inhibited the clone formation ability of MDA-MB-231 and LM2. These results indicated that knockdown of *HTR1D* can strongly inhibit the proliferation and metastasis ability of breast cancer cells in vitro. These experimental results can partially validate the findings obtained from bioinformatics analysis.

## 4. Discussion

As global cancer burdens increase, more efficient biomarkers and novel approaches are urgently needed for cancer patients [35]. Increasing data suggest that *HTGPCRs* are crucial in cancer development and could have therapeutic potential [8]. This study was the first to comprehensively analyze the role of *HTGPCRs* in the development of different tumors, as well as their relationships to these tumors.

Our research revealed that *HTR1D*, *HTR2B*, and *HTR7* were highly expressed in pan-cancer. Specifically, *HTR1D* showed elevated levels in LIHC, LUAD, LUSC, and PAAD, with its increased expression associated with lower overall survival, suggesting that *HTR1D* may function as an oncogenic factor in various cancers. Studies have indicated that *HTR1D* plays a role in the promotion of cell growth by serotonin in human small-cell lung cancer [4] and contributes to the aggressive nature of pancreatic cancer [36], which supports our findings. *HTR2B* was identified as a contributing risk element in COAD, GBMLGG, LUADLUSC, LUSC, STAD, and UVM. Research showed that 32% and 35% of liver cancer patients had *HTR1B* and *HTR2B* expression, respectively, both of which were linked to higher cell growth [17]. Therefore, *HTR2B* can be a potential biomarker for assessing the prognosis of patients. *HTR7* was highly expressed in BLCA, BRCA, ESCA, CESC, KIRC, HNSC, LAML, KIRP, LUSC, PAAD, and PCPG and the high expression of *HTR7* was linked to poor OS in GBMLGG, GBM, LAML, and STAD. The research suggested that *HTR7* served as a negative prognostic biomarker and promoted the growth of triple-negative breast cancer cells [37]. *HTR1A* exhibited low expression across various cancers while elevated levels of *HTR1A* were associated with better overall survival in LGG and GBMLGG. Our earlier research recognized *HTR1A* as a prognostic biomarker for breast cancer patients and *HTR1A* agonists have shown notable antitumor activity in murine models of triple-negative breast cancer [31]. While used in this study, the *HTGPCR* gene family did not show any prognostic significance in breast cancer; this might be due to the heterogeneity of the different subtypes of breast cancer and further research is needed to evaluate the role of the HTRGPCR gene in breast cancer. The prognosis of glioma cancer patients is closely linked to the expression of the *HTGPCR* gene family, yet functional research on *HTGPCRs* in GBM remains scarce and requires thorough investigation. Accordingly, *HTGPCRs* may serve as a prognostic predictive marker or potential cancer predictor.

Cancer is a genetic disease caused by the accumulation of mutations in genes that regulate cell division, survival, invasion, or other characteristics of the transformed phenotype [38]. Genetic mutations connected by inter- and intra-tumor heterogeneity are linked to poor prognosis in some unresectable cancers [39]. Identifying mutational signatures may influence therapy responses. Our results show that the main types of genomic alteration were amplification and deep deletion; the alteration of *HTGPCRs* in pan-cancer patients correlated with poorer prognoses. Alterations in *HTGPCR* genes may play a key role in cancer progression and prognosis.

Moreover, the relationship between *HTGPCR* expression and tumor stages was examined. The expression of *HTR1D* in LUAD, KIRP, and KIRC was significantly higher in Stages I and III while, in LIHC and THCA, the expression in Stage I was relatively lower than in the others. *HTGPCR* expression varied significantly among different stages. PAAD progression involves various serotonin receptor subtypes at distinct tumor phases, with significant *HTR1A* and *HTR1B* expression observed in aggressive PAAD characterized by high Gleason scores and metastatic prostate cancer [40]. Research has revealed a new protective function of serotonin, which facilitates DNA repair during the initial phases of colorectal cancer development [41]. Our findings revealed elevated *HTR1B* levels in advanced pancreatic adenocarcinoma (PAAD) but did not demonstrate a correlation between *HTR1A* and PAAD tumor stages. Extensive further studies are needed to verify the connection between *HTR1A* and tumor progression in PAAD.

Cancer stem cells (CSCs) are proposed to be a key factor in contributing to the diversity within tumors [42,43]. In order to further assess the correlation between tumor heterogeneity and *HTGPCRs*, the Spearman correlation test was performed to assess the correlation between the expression and RNAss and DNAss, TMB, and MSI. In BRCA, the expression of *HTR1A/1F/2B* was negatively related to stemness features. There have been many studies indicating the function of *HTGPCRs* in cancer stemness, especially breast cancer [44] and colorectal cancer [45]. Thus, expression levels of *HTGPCRs* might play a crucial role in maintaining cancer cell stemness properties.

The tumor microenvironment (TME) is a well-organized network comprising cancer cells and various non-cancerous cell types, significantly contributing to immune escape, tumor spread, new blood vessel formation, and cell growth [46]. Our research revealed a strong positive correlation between the expression of *HTR2A/2B/7* and the estimate, stromal, and immune scores. Additionally, the levels of *HTR2A*, *HTR2B*, and *HTR7* were clearly positively associated with the infiltration of immune cells, including B cells, macrophages, and T cells. This implies that *HTR2A/2B/7* could modify the tumor’s microenvironment or influence the level of immune cell infiltration within the tumor. The single-cell analysis also indicated a positive correlation between *HTR2B* and fibroblasts. Numerous research findings indicate that serotonin has intricate interactions with and effects on immune cells, with its receptor engagement playing a vital role in regulating the immune microenvironment [47,48,49]. Additionally, immunotherapy has become the main treatment for different kinds of cancers [50]. Our research identified a notable link between *HTGPCRs* and certain immune checkpoint genes, such as CTLA4, PD-1, and PD-L1, with a particularly strong positive association observed with *HTR1F*, *HTR2A*, *HTR2B*, and *HTR7*. In vitro studies showed that serotonin increased PD-L1 levels in both mouse and human cancer cells through serotonylation [51]. Thus, *HTGPCRs* may have an indirect influence on TME and can be a biomarker for immunotherapy.

In this research, we broadened our investigation to explore the drug responsiveness linked to *HTGPCRs* across various cancer types. We explored the link between *HTGPCR* family gene expression and sensitivity to chemotherapy drugs. High expression of *HTR4* was found to be positively associated with sensitivity to a range of chemotherapy agents. Tegaserod maleate, a partial agonist of *HTR4*, markedly reduced the growth, spread, and migration of breast cancer cells [52]. The levels of *HTR1F* and *HTR7* were negatively associated with the effectiveness of chemotherapy. Research has identified serotonin receptors, specifically *HTR2A*, *HTR2B*, *HTR3A*, and *HTR7*, as potential targets for both the prevention and treatment of stomach cancer [12]. Studies have shown that blocking *HTR1A* receptors and inhibiting serotonin reuptake can suppress the proliferation of distinct prostate cancer cell lines [21]. While numerous studies suggest that drugs acting as agonists or antagonists on 5-HT receptors have antitumor effects, further basic research is required to translate these findings into patient treatments.

A major limitation of this research is its dependence on the bioinformatic examination of publicly available databases to infer information about *HTGPCRs*. Our study reveals the potential of HTRGPCRs as a biomarker for predicting tumor prognosis and treatment response but its role in different tumor subtypes requires further investigation. In this study, we verified the role of *HTR1D* in breast cancer cells, which supports our analysis; additional in vivo and in vitro testing is required to confirm our findings. Moreover, given the important role of the *HTGPCR* gene family in the nervous system and other biological processes, we acknowledge that some *HTGPCR* alterations may overlap with those observed in non-cancerous inflammatory or immune conditions. If *HTGPCR* alterations are not specific to cancer, further research is needed to better understand where these alterations occur and how to differentiate cancer-related changes from those seen in other diseases.

## 5. Conclusions

To conclude, this research offers an extensive cross-cancer examination of the *HTGPCR* gene family, investigating their association with patient outcomes, tumor stemness, the tumor microenvironment, clinical features, and their viability as treatment targets. The high expression of *HTR1D* and *HTR2C* was negatively related to the OS of HNSC, and almost all the *HTGPCR* genes, except *HTR1B* and *HTR4*, were related to the prognosis of glioblastoma. The expression levels of *HTGPCRs* exhibited a strong correlation with RNA and DNA stemness scores, TMB, MSI, as well as stromal and immune scores across various cancer types. *HTR2A*/2B/7 showed significant correlations with immune cells and immune checkpoint genes across various cancers. Moreover, *HTR4* was found to be strongly related to drug sensitivity. Our validation experiment in vitro showed that *HTR1D* knockdown inhibited the metastasis and proliferation of breast cancer cells, which also confirmed part of our analysis.

The *HTGPCR* gene family has been identified as a promising group of biomarkers for diagnosing and predicting the outcomes of different cancers. Their identification could pave the way for enhanced patient outcomes and may offer promising avenues for therapeutic intervention. This research underscores the importance of a comprehensive approach to understanding the intricate interplay between *HTGPCR* genes and cancer biology. By elucidating the roles of these genes, we can better tailor diagnostic and treatment strategies, ultimately contributing to the advancement of personalized medicine in oncology.

## Figures and Tables

**Table 1:** Survival analysis of *HTGPCR* expression in pan-cancer from different databases. The clinical outcome is overall survival (OS).

Gene	Role	Log-Rank Analysis	Cox Analysis	KM Plotter
***HTR1A***	**Protective**	LGG/GBMLGG	LGG	-
	**Detrimental**	-	SARC	-
***HTR1B***	**Protective**	ESCC/KIRC/READ	READ	-
	**Detrimental**	KIRP/MESO	MESO	KIRP
***HTR1D***	**Protective**	-	-	-
	**Detrimental**	BLCA/GBMLGG/HNSC/LGG/ LIHC/LUAD/LUADLUSC/ PAAD/SARC	ACC/HNSC/KIRC/KIRP/ LGG/LIHC/LUAD/MESO /OV/PAAD	LIHC/HNSC/KIRC/LUAD/ SARC
***HTR1E***	**Protective**	GBMLGG	-	BLCA
	**Detrimental**	-	THCA	UCEC
***HTR1F***	**Protective**	-	-	-
	**Detrimental**	LAML/LGG /GBMLGG/ MESO/STAD	ACC/KICH/LAML/STAD	KIRP/STAD
***HTR2A***	**Protective**	GBMLGG/LGG	LGG	-
	**Detrimental**	KICH/LUSC	KICH/STAD	STAD
***HTR2B***	**Protective**	ESAD/ESCA/LUAD/PCPG/ UCEC	LAML/	ESAD
	**Detrimental**	COAD/GBMLGG/ LUADLUSC/LUSC/ STAD/UVM	BLCA/KIRP/LGG/OV/ UVM	LUSC/STAD
***HTR2C***	**Protective**	GBM/GBMLGG	LGG	-
	**Detrimental**	HNSC/STAD	ACC//CHOL/COAD/HNSC/ KICH/KIRC/LAML/ THCA/UCEC	HNSC/KIRC/LIHC/THCA
***HTR4***	**Protective**	BLCA/LUAD/SKCM	BLCA/SKCM	BLCA/LUAD/PAAD
	**Detrimental**	-	LGG/THCA	THYM
***HTR5A***	**Protective**	GBMLGG	-	-
	**Detrimental**	-	LIHC/SARC/SKCM/UCEC	ESAD/LIHC
***HTR5BP***	**Protective**	LUADLUSC	-	-
	**Detrimental**	LGG	ACC/LICH/LGG/UCEC	-
***HTR6***	**Protective**	GBMLGG/LGG	HNSC/LGG/	-
	**Detrimental**	ACC/GBM/KIRP/MESO/UCEC	UCEC	UCEC
***HTR7***	**Protective**	KICH	-	KIRC
	**Detrimental**	GBMLGG/GBM/LAML/STAD	GBM/HNSC/KICH/LAML/ MESO/OV/STAD/UVM	-

## Data Availability

Publicly available datasets were analyzed in this study. These data can be found here: 33 types of cancers from the Xena browser (https://xena.ucsc.edu/, accessed date 17 July 2021) and the external validation cohort from the GEO database (https://www.ncbi.nlm.nih.gov/geo/, accessed date 28 May 2024).

## Supplementary Materials

The following supporting information can be downloaded at: https://www.mdpi.com/article/10.3390/genes15121541/s1. Figures S1 and S2: Survival analysis of 33 cancer types using *HTGPCRs*; Figure S3: The correlation between *HTGPCRs* and OS by univariate Cox regressions in pan-cancer; Figure S4: Overall survival curves comparing the high and low expression of *HTGPCRs* in various cancers from the Kaplan–Meier Plotter database; Figure S5: Copy number alternations associated with *HTGPCR* expression in cBioPortal; Figure S6: Association between the expression of the *HTGPCR* family and the clinical stage across 33 cancer types; Figure S7: Single-cell analysis. Table S1: The detailed information of TCGA pan-cancer data.
