# Supplementary material for: 5-Hydroxytryptamine G-Protein-Coupled Receptor Family Genes: Key Players in Cancer Prognosis, Immune Regulation, and Therapeutic Response
Source: Genes (Basel). 2024 Nov 28;15(12):1541. doi: 10.3390/genes15121541 (PMC11675146; doi:10.3390/genes15121541)
Supplement: Supplementary file 1 [file genes-15-01541-s001.zip › genes-3315146-supplementary.pdf]

Supplementary Material

1. Supplemental Tables

Table S1 The detailed information of TCGA pan-cancer data

Cancer abbreviation	Tumor types	Tumor samples	Normal samples	Total samples
**ACC**	Adrenocortical carcinoma	77	0	77
**BLCA**	Bladder Urothelial Carcinoma	407	19	426
**BRCA**	Breast invasive carcinoma	1092	113	1205
**CESC**	Cervical squamous cell carcinoma and endocervical adenocarcinoma	304	3	307
**CHOL**	Cholangiocarcinoma	36	9	45
**COAD**	Colon adenocarcinoma	288	41	329
**COADREAD**	Colon adenocarcinoma/Rectum adenocarcinoma Esophageal carcinoma	380	51	431
**DLBC**	Lymphoid Neoplasm Diffuse Large B-cell Lymphoma	181	13	194
**ESCA**	Esophageal carcinoma	47	0	47
**GBM**	Glioblastoma multiforme	153	5	158
**GBMLGG**	Glioma	662	5	667
**HNSC**	Head and Neck squamous cell carcinoma	518	44	562
**KICH**	Kidney Chromophobe	66	129	195
**KIRC**	Kidney renal clear cell carcinoma	530	129	659
**KIRP**	Kidney renal papillary cell carcinoma	288	129	417
**LAML**	Acute Myeloid Leukemia	509	5	514
**LGG**	Brain Lower Grade Glioma	173	0	173
**LIHC**	Liver hepatocellular carcinoma	369	50	419
**LUAD**	Lung adenocarcinoma	513	109	622
**LUSC**	Lung squamous cell carcinoma	498	109	607
**MESO**	Mesothelioma	178	4	182
**OV**	Ovarian serous cystadenocarcinoma	177	3	180
**PAAD**	Pancreatic adenocarcinoma	87	0	87
**PCPG**	Pheochromocytoma and Paraganglioma	495	52	547
**PRAD**	Prostate adenocarcinoma	419	0	419
**READ**	Rectum adenocarcinoma	92	10	102
**SARC**	Sarcoma	258	0	258
**STAD**	Stomach adenocarcinoma	414	36	450
**SKCM**	Skin Cutaneous Melanoma	102	0	102
**STES**	Stomach and Esophageal carcinoma	595	49	644
**TGCT**	Testicular Germ Cell Tumors	148	0	148
**THCA**	Thyroid carcinoma	504	59	563
**THYM**	Thymoma	119	0	119
**UCEC**	Uterine Corpus Endometrial Carcinoma	180	23	203
**UCS**	Uterine Carcinosarcoma	57	0	57
**UVM**	Uveal Melanoma	79	0	79

2. Supplemental figures

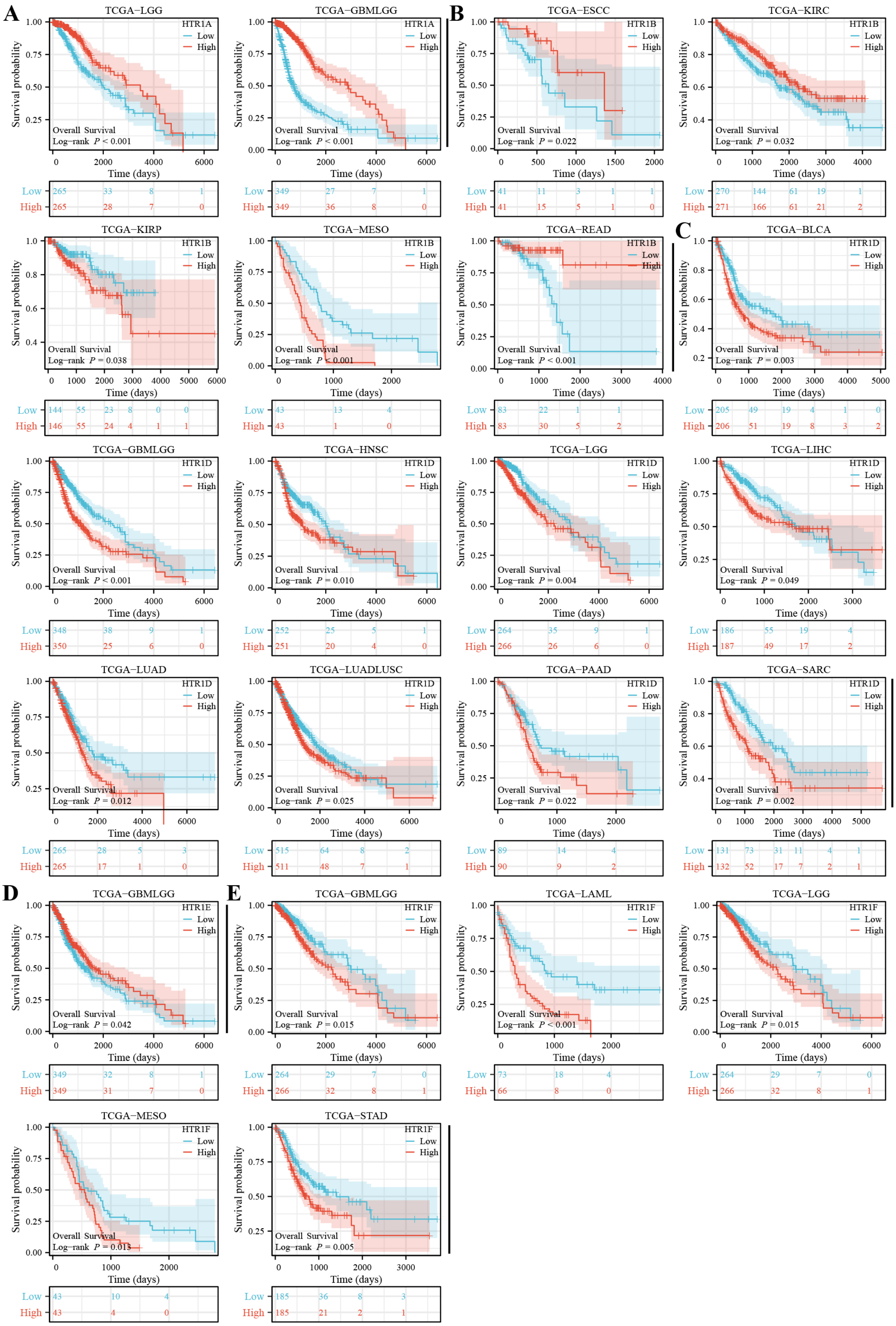

Figure S1. Survival analysis of 33 cancer types using HTGPCRs. (A) HTR1A (B) HTR1B (C) HTR1D (D) HTR1E (E) HTR1F.

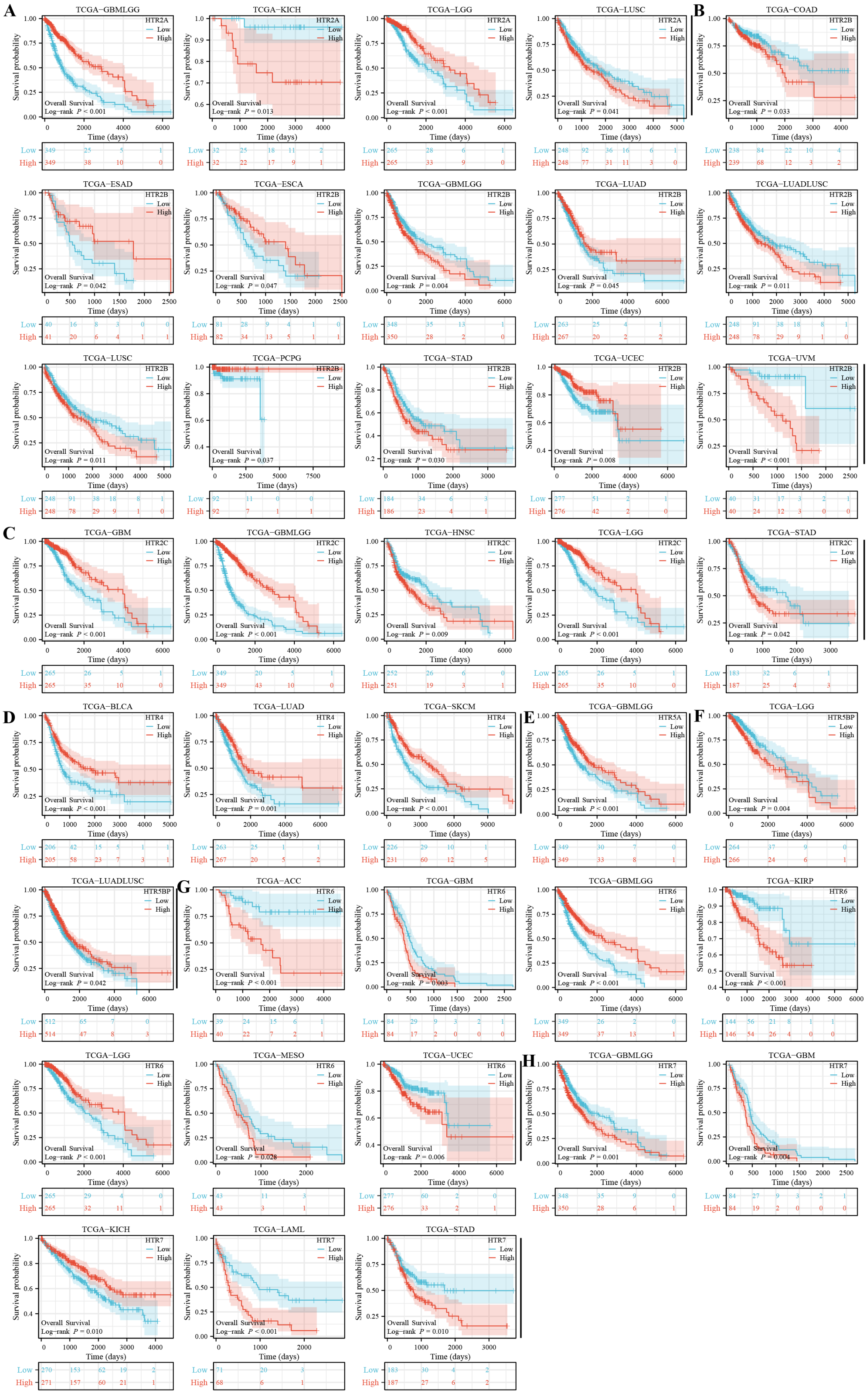

Figure S2. Survival analysis of 33 cancer types using HTGPCRs. (A) HTR2A (B) HTR2B (C) HTR2C (D) HTR4 (E) HTR5A (F) HTR5BP (G) HTR6 (H) HTR7.

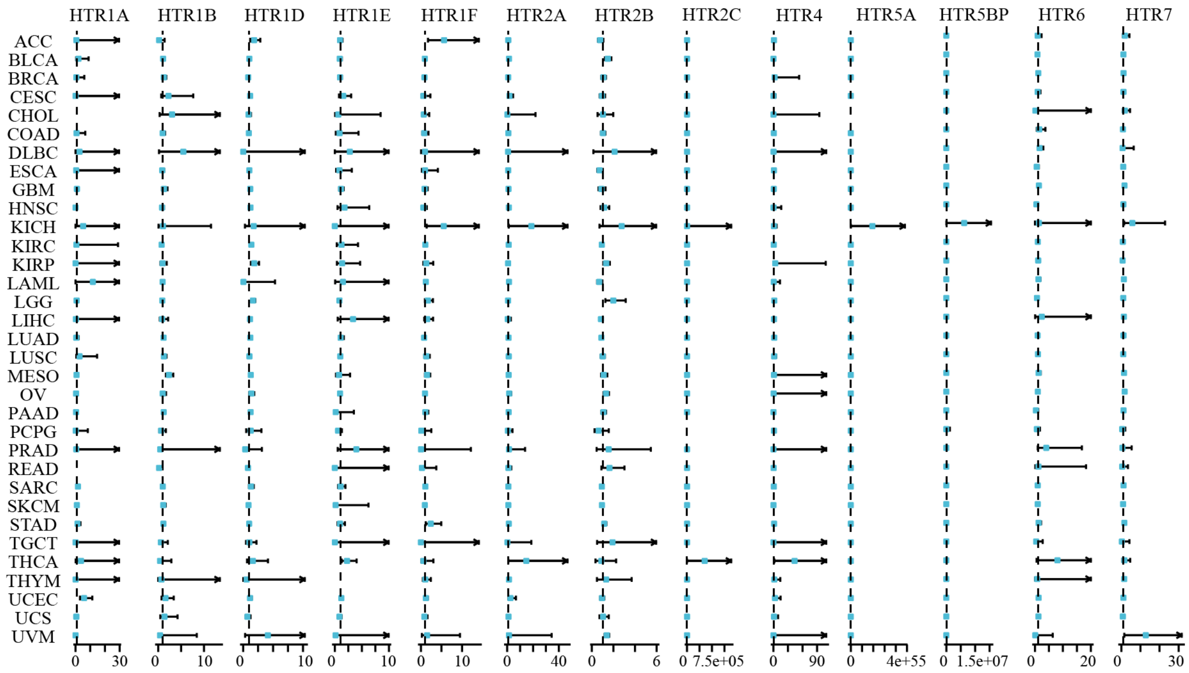

Figure S3. The correlation between HTGPCRs and OS by univariate Cox regressions in pan-cancer. A hazard ratio less than 1 indicates a lower risk, while a hazard ratio greater than 1 indicates a higher risk.

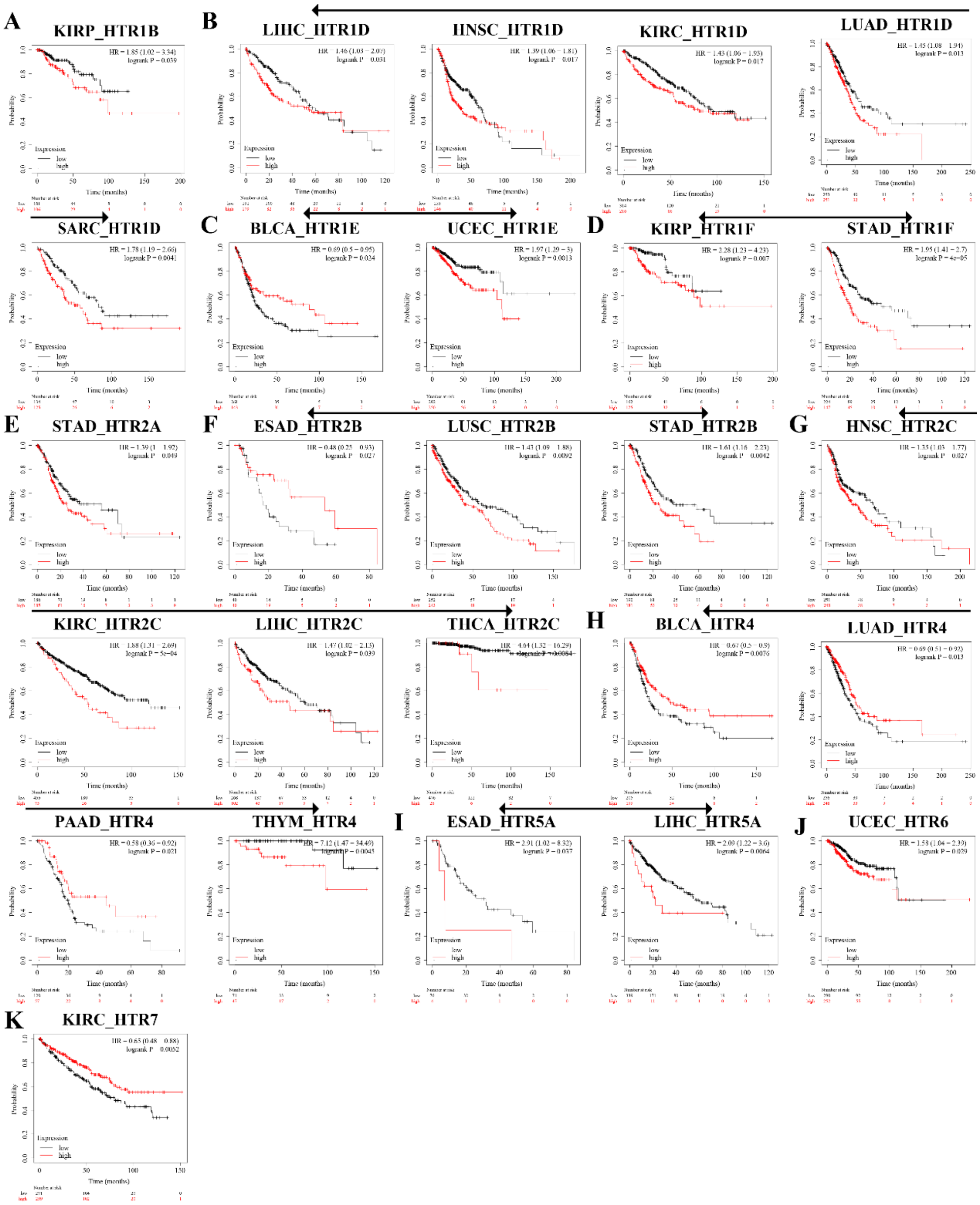

Figure S4. Overall survival curves comparing the high and low expression of HTGPCRs in various cancers from Kaplan-Meier Plotter database. (A) HTR1B (B) HTR1D (C) HTR1E (D) HTR1F (E) HTR2A (F) HTR2B (G) HTR2C (H) HTR4 (I) HTR5A (J) HTR6 (K) HTR7.

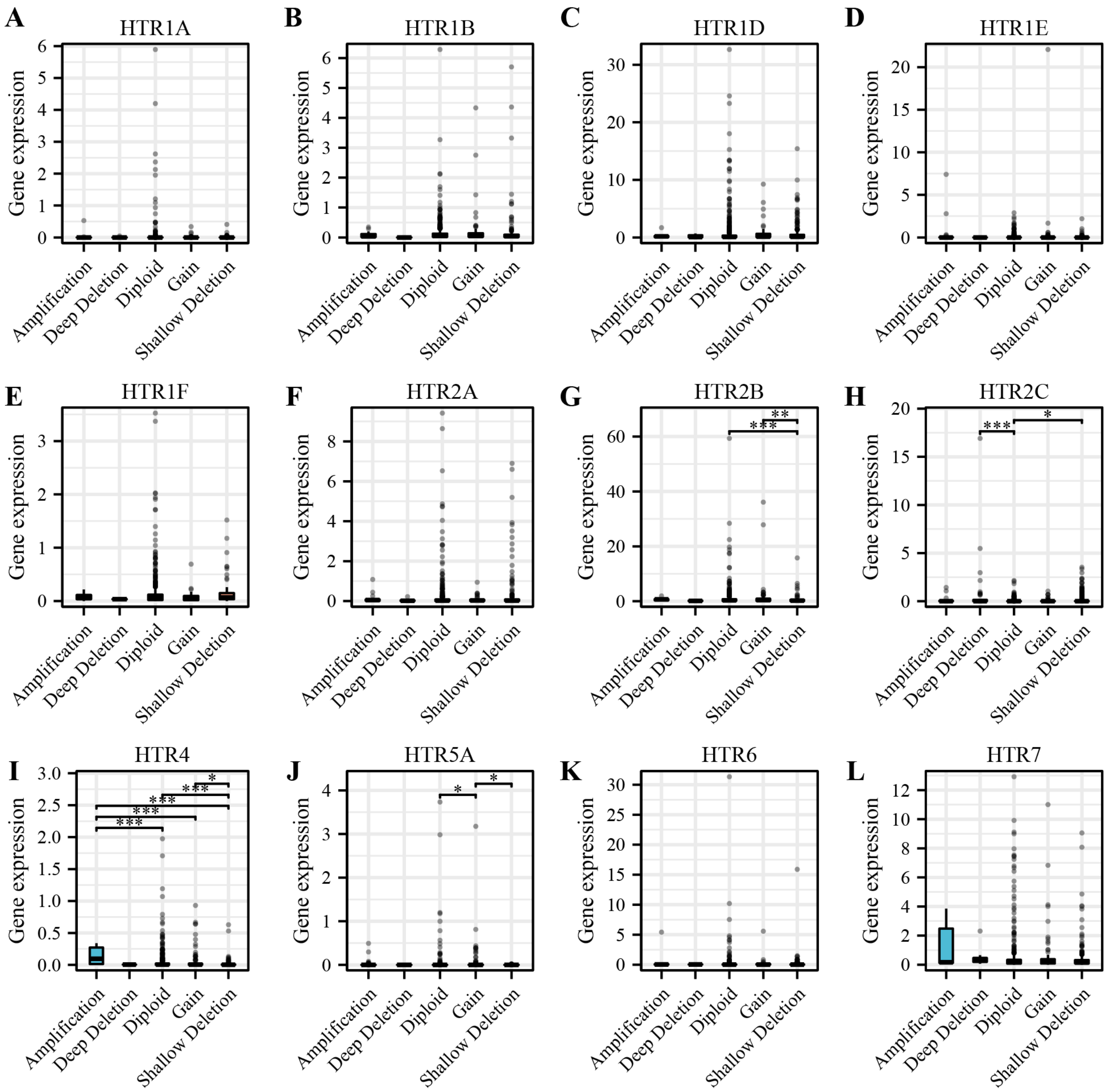

Figure S5. Copy number alternations associated with HTGPCRs expression in cBioPortal. (A-L) HTR1A (B) HTR1B (C) HTR1D (D) HTR1E (E) HTR1F (F) HTR2A (G) HTR2B (H) HTR2C (I) HTR4 (J) HTR5A (K) HTR6 (L) HTR7. *P < 0.05, **P < 0.01, **P < 0.001.

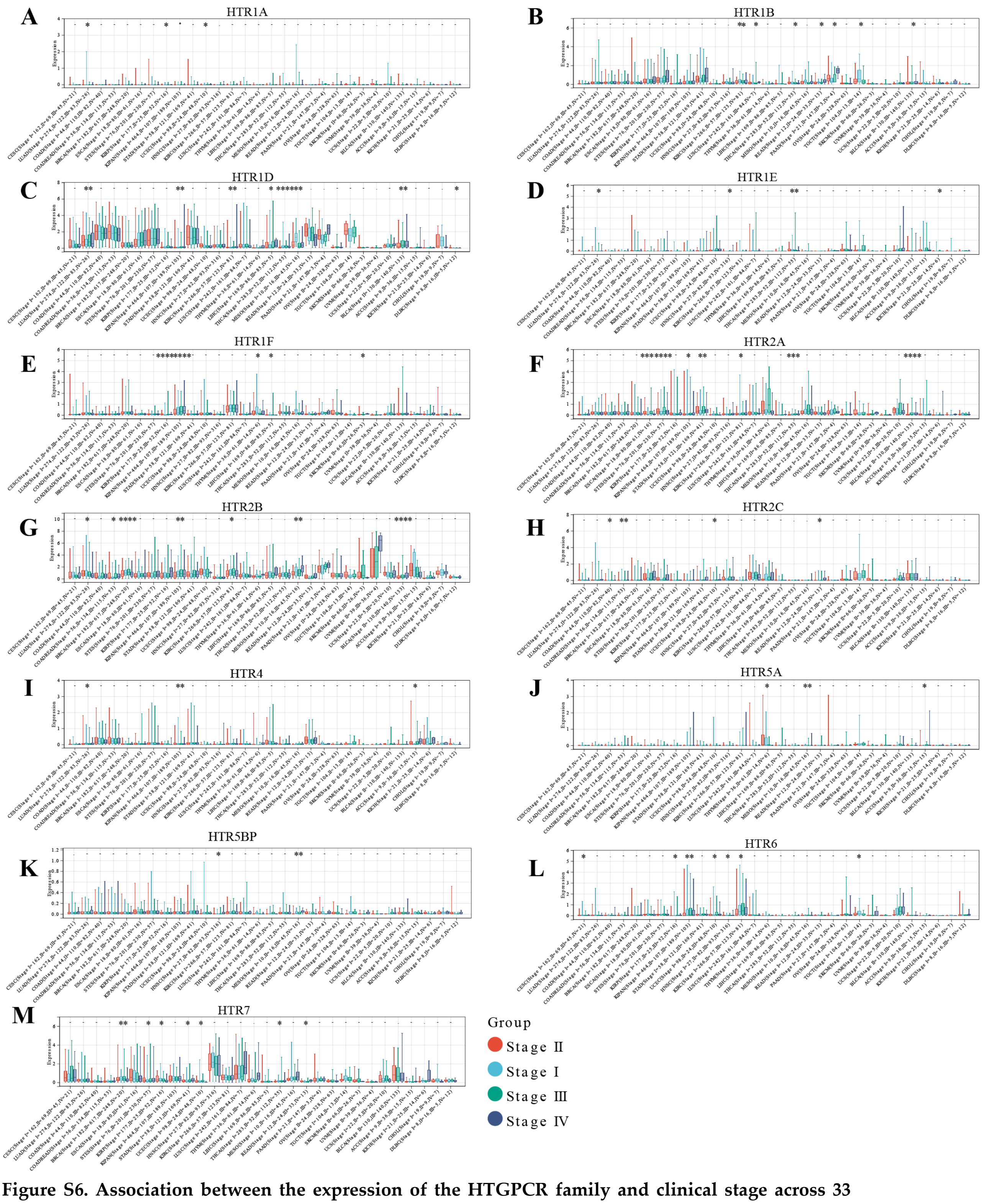

Figure S6. Association between the expression of the HTGPCR family and clinical stage across 33 cancer types (A-M). *P < 0.05, **P < 0.01, **P < 0.001.

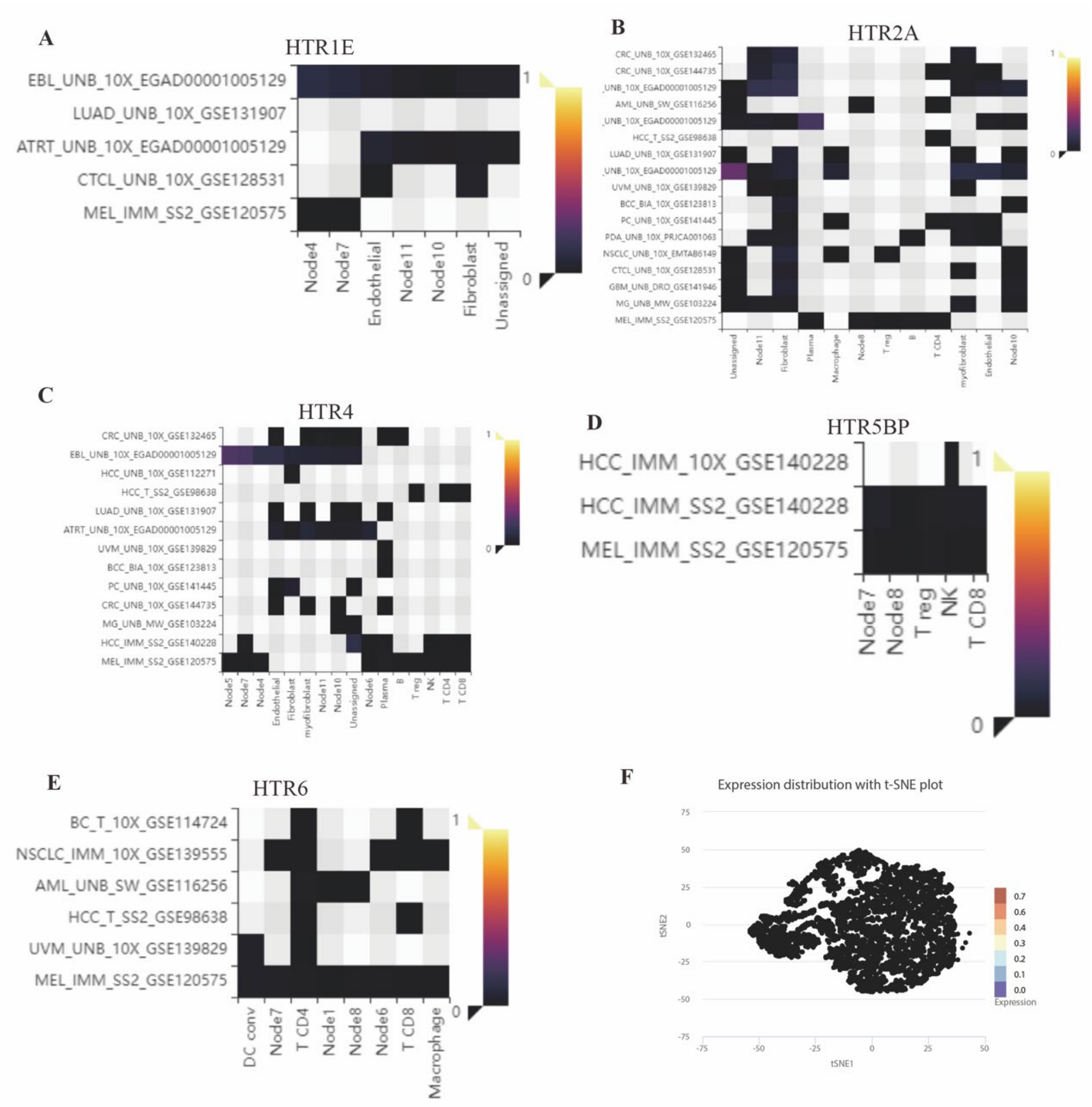

Figure S7. Single-cell analysis. The expression distribution of HTR1E (A), HTR2A (B), HTR4 (C), HTR5BP (D), and HTR6 (E) at the single-cell level. (F) The expression distribution of the gene set of HTGPCRs with t-SNE plot in RB is displayed.
